# Stability analysis of the GAL regulatory network in *Saccharomyces cerevisiae* and *Kluyveromyces lactis*

**DOI:** 10.1186/1471-2105-11-S1-S43

**Published:** 2010-01-18

**Authors:** Vishwesh V Kulkarni, Venkatesh Kareenhalli, Pushkar Malakar, Lucy Y Pao, Michael G Safonov, Ganesh A Viswanathan

**Affiliations:** 1 Department of Electrical Engineering, Indian Institute of Technology Bombay, Mumbai 400 076, India; 2 Department of Chemical Engineering, Indian Institute of Technology Bombay, Mumbai 400 076, India; 3 Department of Electrical, Computer, and Energy Engineering, University of Colorado, Boulder, CO 80302, USA; 4 Department of Electrical Engineering, University of Southern California, Los Angeles, 90089-2563, USA

## Abstract

**Background:**

In the yeast *Saccharomyces cerevisiae*, interactions between galactose, Gal3p, Gal80p, and Gal4p determine the transcriptional status of the genes required for the galactose utilization. Increase in the cellular galactose concentration causes the galactose molecules to bind onto Gal3p which, via Gal80p, activates Gal4p, which induces the GAL3 and GAL80 gene transcription. Recently, a linear time-invariant multi-input multi-output (MIMO) model of this GAL regulatory network has been proposed; the inputs being galactose and Gal4p, and the outputs being the active Gal4p and galactose utilization. Unfortunately, this model assumes the cell culture to be homogeneous, although it is not so in practice. We overcome this drawback by including more biochemical reactions, and derive a quadratic ordinary differential equation (ODE) based model.

**Results:**

We show that the model, referred to above, does not exhibit bistability. We establish sufficiency conditions for the domain of attraction of an equilibrium point of our ODE model for the special case of full-state feedback controller. We observe that the GAL regulatory system of *Kluyveromyces lactis *exhibits an aberration of monotone nonlinearity and apply the Rantzer multipliers to establish a class of stabilizing controllers for this system.

**Conclusion:**

Feedback in a GAL regulatory system can be used to enhance the cellular memory. We show that the system can be modeled as a quadratic nonlinear system for which the effect of feedback on the domain of attraction of the equilibrium point can be characterized using *linear matrix inequality *(LMI) conditions that are easily implementable in software. The benefit of this result is that a mathematically sound approach to the synthesis of full-state and partial-state feedback controllers to regulate the cellular memory is now possible, irrespective of the number of state-variables or parameters of interest.

## Background

### Introduction to the GAL regulatory system

**Figure 1:**
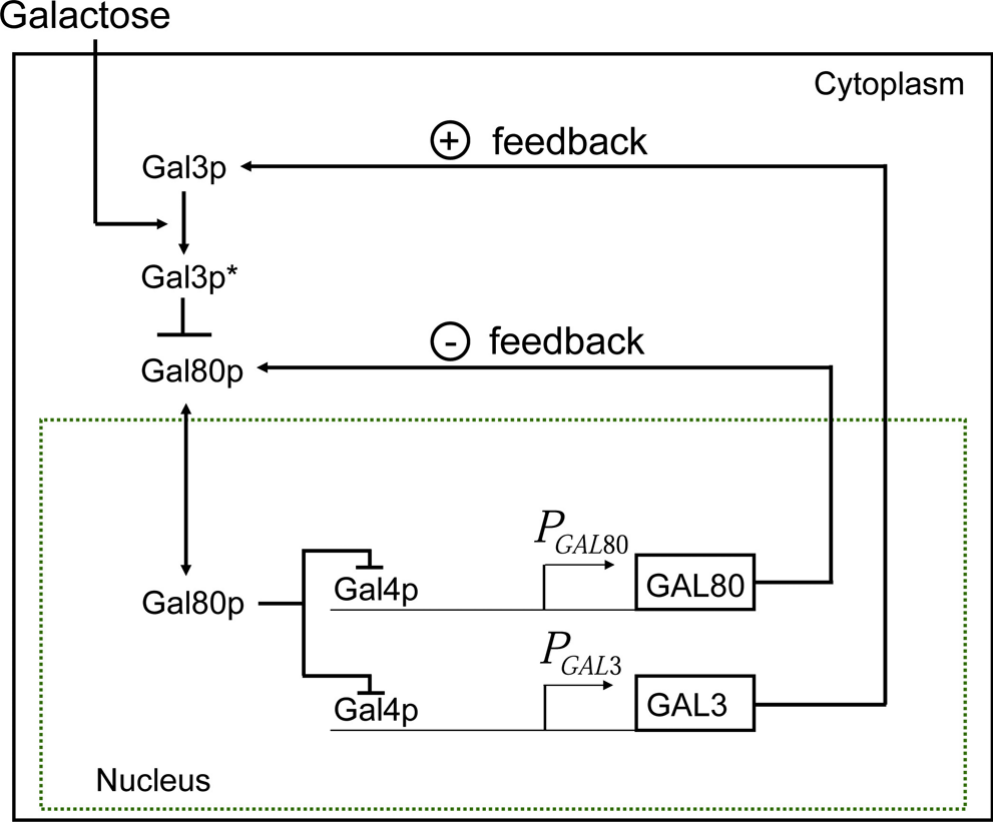
**The galactose signalling pathway**. The external galactose signal controls the transcriptional activity of the GAL genes. Galactose can shuttle between the cytoplasm and the nucleus. The galactose bound stage of the protein Gal3p is Gal3p*. The pointed arrows indicate activation whereas the blunt arrows indicate inhibition.

Naturally occurring networks of genes and proteins, especially in eukaryotic organisms, feature multiple complex nested feedback loops. So, although gene expressions can be affected at many levels including protein-DNA interactions, protein-protein interactions, and protein-small molecule interactions, it is difficult to characterize, a priori, the systemic effect of these changes. An example of such networks is the galactose signalling pathway in the yeast *Saccharomyces cerevisiae*. Despite extensive data on its molecular interactions, an a priori prediction of its systemic behavior remains challenging (see [1,2], and [3]). In the GAL regulatory network (see Fig. 1), the galactose signal propagates through a four-stage signalling cascade. At the uppermost stage is Gal2p, which imports extracellular galactose into the cell.

Subsequently, intracellular galactose binds to and activates Gal3p (see [2] and [3]). At the third stage of this cascade, the activated Gal3p binds to and sequesters Gal80p in the cytoplasm, depleting Gal80p from the nucleus. The transcriptional activator Gal4p, which is constitutively bound to promoters of the GAL genes, is then released from the inhibitory action of Gal80p and activates expression of genes at the output of the cascade, including GAL1, GAL2, GAL3 and GAL80. Because an increase in Gal2p and Gal3p concentration results in enhanced transcriptional activity, these two proteins each enforce a positive feedback loop whereas Gal80p enforces a negative feedback loop (see [1]).

### Modelling assumptions

We mostly follow [1] to model the GAL regulatory network. We denote genes in all capital letters, and proteins with only first letter in capital letters. We focus on only early stages of the galactose induction, and disregard the events that occur after the Gal4p phosphorylation. We overlook the details of signal transmission from galactose to Gal4p. In other words, Gal4p encountered in our model could be bound to DNA or could be bound to DNA and Gal80p. Likewise, Gal80p in our model is either bound to DNA and Gal4p or bound to Gal3p or unbound. Gal1p and Gal3p are taken to play a similar role, and are together referred to as Gal3p.

### Modelling the GAL regulatory system

**Figure 2:**
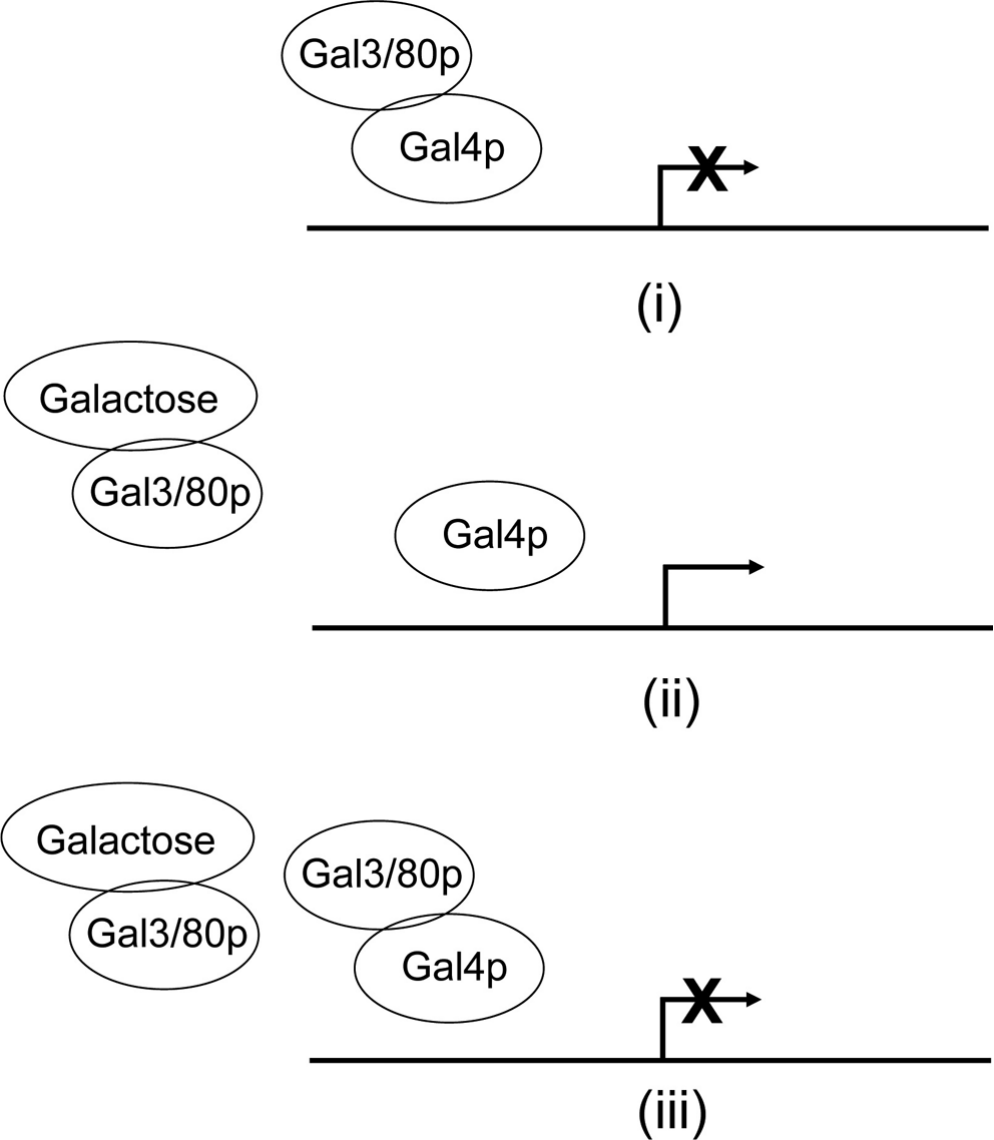
**The galactose induction loop**. This figure is reproduced from [4]. (i) In the absence of galactose, the transcriptional activity of Gal4p is inhibited by Gal3/80p. (ii) The association of galactose with Gal3/80p allows Gal4p to be freed from Gal80p inhibition and to activate the transcription of new Gal3/80p. (iii) Newly synthesized Gal3/80p inhibits the transcriptional activity of Gal4p.

We shall first summarize the logic of [1] and describe its model of the GAL regulatory system, and then relax some of its assumptions to derive a nonlinear model. The states of interest of the system are shown in Fig. 2. In the absence of galactose, Gal4p can bind to Gal80p and has no transcriptional activity. Following a step increase in galactose, Gal3p rapidly binds galactose, and Gal80p is consumed by being recruited in a complex with Gal3p. As the concentration of the unbound Gal80p decreases, the Gal4p/80p complex is destabilized, which activates Gal4p. Activated Gal4p then initiates the slower biosynthetic reactions, viz., transcription of the GAL genes including GAL3 and GAL80, followed by translation into their protein products. Following Gal4p activation, and consequent GAL gene expression, newly synthesized Gal3p and Gal80p shift the equilibrium back towards Gal4p inactivation. As a result, GAL transcriptional activity decreases. Newly formed proteins can bind to the incoming galactose molecules, thus restoring sensitivity to any further galactose input. This effectively closes the feedback loop. We lump Gal3p and Gal80p together as the Gal3/80p complex. In the absence of galactose, Gal3p, Gal80p and Gal4p form an inactive complex Gal4/3/80p called receptor R. A bound receptor BR comprising Gal3p, Gal80p and galactose remains inactive or may be degraded. The total Gal4p concentration is assumed to be constant during the GAL response, as suggested by transcriptomics data (see [4,3], and [5]).

### Biochemical reactions in the GAL regulatory system

We represent a gene, its encoded mRNA and protein as a single entity. G4 denotes Gal4p protein and  denotes activated Gal4p protein. The reactions of interest are as follows:(1)

The first reaction is the slow biosynthesis of transcription and translation, comprising the binding of Gal4p to the GAL3 and GAL80 gene promoters, and all subsequent actions until Gal3/80p molecules are newly synthesized one at a time, and Gal4p stays activated. The second reaction is the inactivation of Gal4p into its inactive form called the receptor R, and the third reaction is the activation of Gal4p due to the binding of galactose, Gal, to the receptor, yielding the bound receptor, BR. For simplicity, these three reactions are reduced to the following two reactions in [4]:(2)

Let *S*_0 _denote the initial quantity of galactose and let *R*_0 _denote the initial quantity of Gal4p. Let us normalize *K*_1 _to unity. Then, it is shown in [4] that the above model of the GAL regulatory system gives rise to a system of differential equations that can be analyzed using the phase-plane method to better understand how the GAL regulatory system is robust to parameter variations and gene transcription time-delays. In deriving this model, [4] makes the following assumptions which may not hold in practice: (i) the cell culture has a homogeneous distribution whence *K*_*i *_are equal; and (ii) the feedback loops of GAL3 and GAL80 can be lumped together. In this paper, we relax the above two assumptions and derive a less simplified nonlinear model. We then apply multiplier theory to better understand stability and robustness of the GAL regulatory network.

## Methods

### A nonlinear state-space model of the GAL regulatory system

The state-space model derived in [4] is as follows. Let *R *and Gal be the states *x*_1 _and *x*_2 _of the system, and let *x *≐ [*x*_1 _*x*_2_]^*T*^. Define *α*_1 _= -(*K*_3_*S*_0 _+ 1), *α*_2 _= -*K*_3_*R*_0_, *α*_3 _= - *K*_3_*S*_0 _where *K*_*i *_are the kinetic reaction constants, and the nonlinearity *f*(*ζ*_1_, *ζ*_2_) = (*K*_3 _- *K*_2_) *ζ*_1_*ζ*_2_. Then, a state-space model of the GAL system is , where

**Remark 1 **In [4], only the initial condition response, i.e., the response to *S*_0 _and *R*_0_, is considered. The two inputs of interest are the galactose injected in the cell, and *R*; the first input can be varied using Gal2p, and the second input can be varied by transforming Gal4 deleted cells with a plasmid expressing Gal4.

**Remark 2 **Arguing that *K*_*i *_are all equal, Φ(*x*) is set to zero in [4], and the phase-plane method is applied on the linearized 2-state system to determine the conditions under which the system is stable and robust to the gene expression delays. In practice, however, the cells are not uniformly distributed whence *K*_*i *_are not equal so that the nonlinearity Φ cannot be neglected. Further, as the following lemma shows,  fails to exhibit bistability, a key property of the GAL regulatory network, even after Φ is accounted for.

**Lemma 1 ***has a unique steady state and does not exhibit a Hopf bifurcation*.

**Proof: **See Additional file 1.

**Remark 3 **Lemma 1 implies that the GAL regulatory system model of [4] is not bistable. However, it is well known that the GAL regulatory system exhibits bistability (see [1]). This anomaly results because, in deriving , the nonlinear feedback loops of GAL3 and GAL80, one of which is positive whereas the other is negative, are overly simplified using a single negative feedback loop in [4]. We propose a correction by including more molecular reactions and, hence, more state variables in our model.

Let us choose the state variables *x*_1 _= , *x*_2 _= *Gal*3/80, *x*_3 _= *R*, *x*_4 _= *BR*, *x*_5 _= *Gal*, and let *x *≐ [*x*_1 _*x*_2 _*x*_3 _*x*_4 _*x*_5_]^*T *^. Then (1) can be expressed as  = *Ax *+ Φ(*x*) + *Bu*, where(3)

where *a*_*i *_and *b*_*i *_are the kinetic reaction constants, *ζ*_*i *_are the degradation rates, and *u *is the input galactose. This is our model  of the GAL regulatory system. Note that the nonlinearity Φ(*x*) is quadratic and can be expressed as Φ(*x*) = *x*^*T*^**N***x *where **N **≐ [*N*_1 _*N*_2_... *N*_5_]^*T *^for some *N*_*i *_∈ ℝ^5×5^. Literature on the stability analysis of such systems is sparse although sufficiency conditions have been established in [6]. It appears that ℒ_2 _stability cannot be expected of multistable models due to the following reason.

**Lemma 2 ***A bistable controllable state-space system is not *ℒ_2_-*stable*.

**Proof: **Let *u*, *x *denote the input and output of the system. Since the system is bistable, there exists a time *τ *and control signals *u*_1_, *u*_2 _∈ *P*_*τ *_ℒ_2 _that drive the system output to each of two distinct constant equilibrium output values, say *x*_1*o *_and *x*_2*o*_, at time *τ *such that *x*_1_(*t*) = *x*_1*o *_and *x*_2_(*t*) = *x*_2*o *_for all *t *≥ *τ *. Hence, *u*_1_, *u*_2 _∈ ℒ_2_, but *x*_1 _- *x*_2 _∉ ℒ_2_. Therefore, either *x*_1 _∉ ℒ_2 _or *x*_2 _∉ ℒ_2 _or both. QED.

As a result, we focus only on establishing a domain of attraction for an equilibrium point of such models. Determination of the domain of attraction is useful since it determines the stability region for cellular memory that can be controlled using a linear feedback of the gene expression states. An extreme example is that of persistent memory, obtained by deleting the GAL80 feedback loop; this phenomenon is observed in mutant genes [1].

**Remark 4 **Experimentally, we have observed that the input-output map of *Kluyveromyces lactis *with GAL80 as the output and galactose as the input is an aberration of friction nonlinearity. Multiplier theoretic stability analysis results (see [7-10], and [11]) can be applied to determine the finite-gain stability of such reduced order models as we demonstrate in the Results section.

### Stability and multipliers

**Figure 3:**
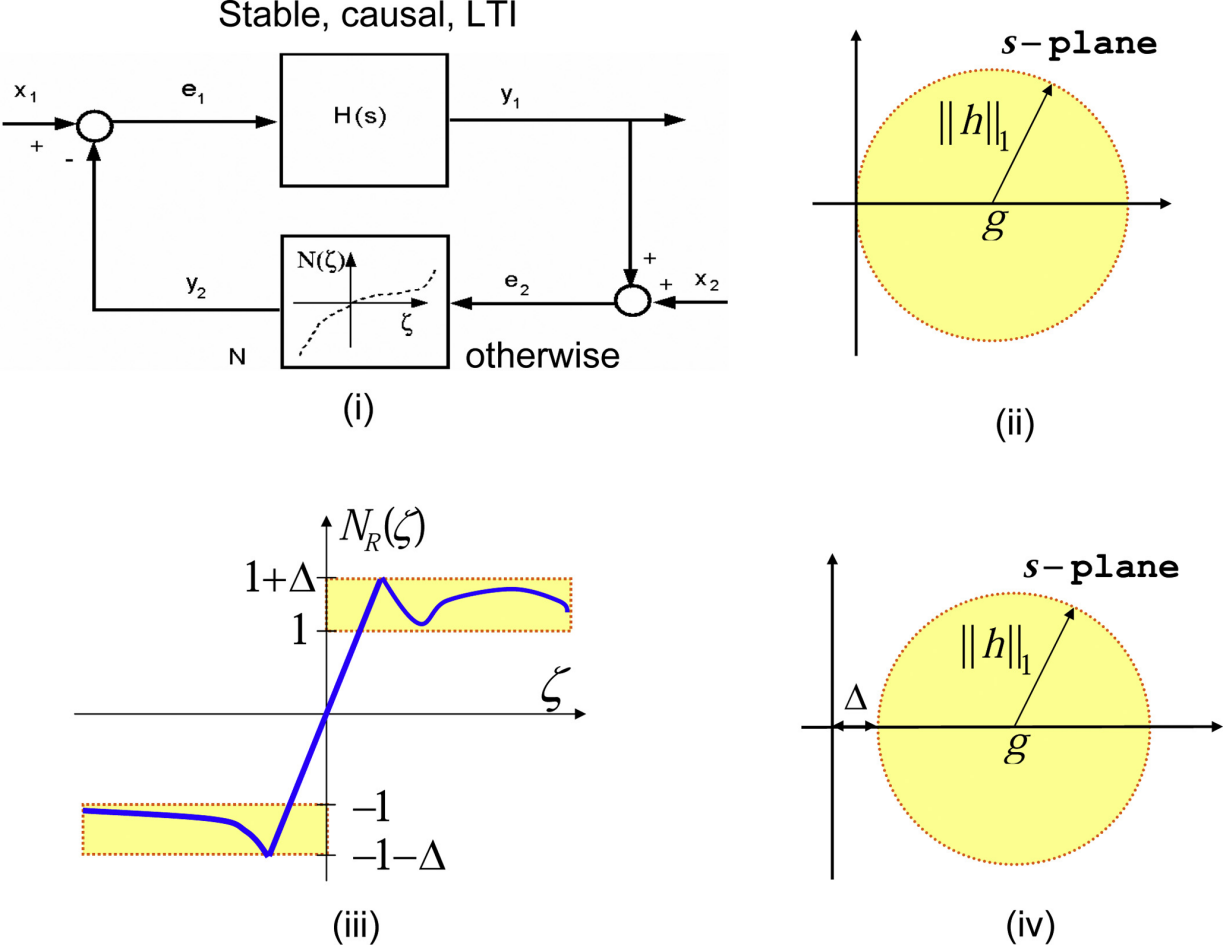
**Stability analysis of feedback systems**. (i) We decompose any given system as a feedback interconnection of a linear time-invariant system *H *and an otherwise system *N*. Stability of the feedback interconnection follows if there exists a hyperplane that separates the graph of *H *from the inverse graph of *N*. If *N *is a monotone nonlinearity, the Zames-Falb multipliers are commonly used to reduce conservatism in multiplier-based stability analysis of this system. (ii) The Nyquist plot of a Zames-Falb multiplier is constrained to lie inside an open disc in the right-half *s*-plane. (iii) Rantzer has investigated these distortions of monotone nonlinearities, and has shown that the stability multipliers for such systems can be obtained by adding a DC offset to the Zames-Falb multipliers (see [15]). The Nyquist plot of these multipliers is constrained to lie in the open disc shown in (iv).

We now formally introduce the notation and the notion of stability; a detailed description of these notions is available in [9] and [10]. Let (ℝ^+^) ℝ denote the set of all (nonnegative) real numbers. Let (·)' (or (·)^*T*^) denote the transpose of a vector or a matrix (·). Let the inner-product  and let the norm . The vector space ℒ_2 _comprises all signals *x *for which ||*x*|| < ∞. The norm . The Dirac delta function is denoted *δ*(·). The time-truncation operator is denoted *P*_*τ*_. In stability analysis, a given system  is often decomposed into two interconnected subsystems -- a *linear time-invariant *(LTI) subsystem  in the feedforward path and an otherwise subsystem  in the feedback path (see Fig. 3(i)). Stability of  is then deduced if there exists a quadratic functional that separates the graph of  from the inverse graph of  (see [12]). Certain classes of convolution operators, also called *stability multipliers *(see [7]), specify such functionals. The larger the class of the stability multipliers, the lower the conservatism in the stability analysis [13]. Stability multipliers for memoryless monotone nonlinearities are the Zames-Falb multipliers [8] and their limiting cases include Popov multipliers [11] and RL/RC multipliers [14]. A key property of such a multiplier *M *is that it preserves the positivity of a memoryless monotone nonlinearity *N *in the sense that the positivity of *N *implies the positivity of *MN*. Well known examples of positivity preserving multipliers include the Popov multipliers and the Zames-Falb multipliers (see [7,8], and [[9], Chapter 3]).

**Definition 1 ***A system **mapping u *∈ ℒ_2 _*into y *∈ ℒ_2 _*is said to be *finite gain stable *if there exists γ *≥ 0 *such that*||(*u*)|| ≤ *γ *||*u*|| *for all u *∈ ℒ_2_.

**Definition 2 ***The class **of *monotone nonlinearities *consists of all memoryless mappings N *: ℝ^*n *^↦ ℝ^*n *^*such that: *(*i*)*N is the gradient of a convex real-valued function, and (ii) there exists C *∈ ℝ^+ ^*s.t*. ||*N *(*x*)|| ≤ *C*||*x*|| ∀ *x *∈ ℒ_2_. *The class *.

**Definition 3 ***The class *ℳ_*ZF *_*of Zames-Falb multipliers denotes the class of convolution operators, either continuous-time or discrete-time, such that the impulse response of an M *∈ ℳ_*ZF *_*is of the form m*(·) = *g δ *(·) + *h*(·) *with *||*h*||_1 _<*g*, *h*(*t*) ≤ 0 ∀ *t*, *where g*, *h*(·) ∈ ℝ.

**Figure 4:**
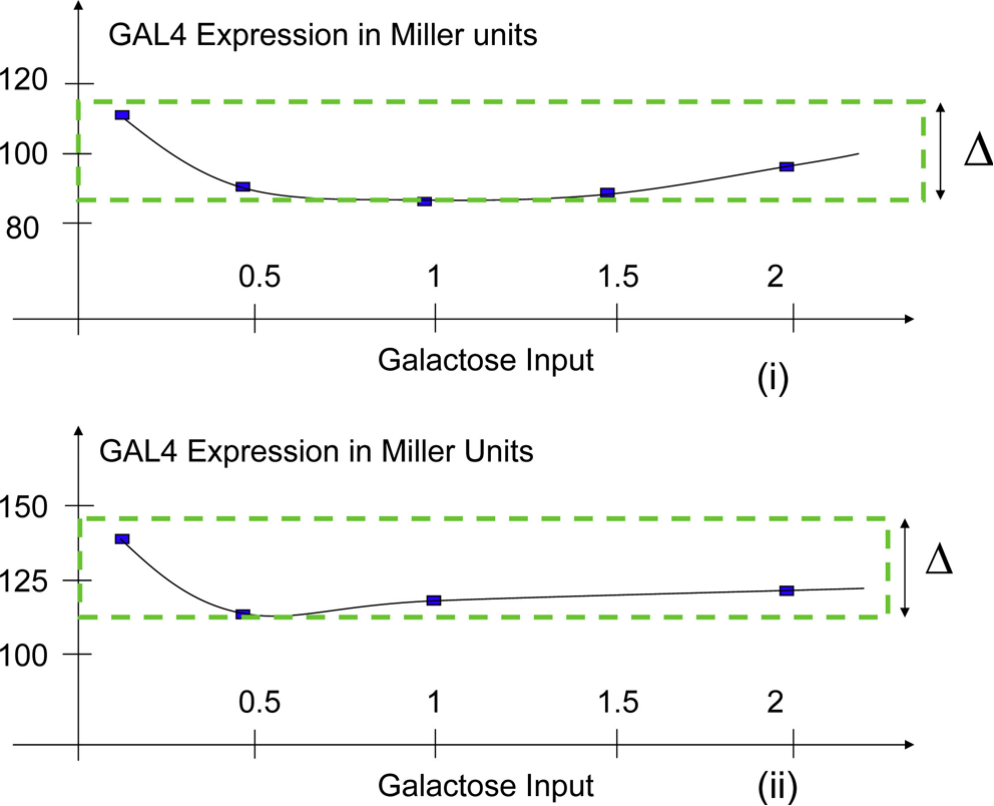
**nonmonotone nonlinearity in Kluveromycetes lactis**. We experimentally observed that the synthesis of  using galactose in *Kluveromycetes lactis *exhibits the shown nonlinearity. WT culture was grown in different concentrations of lactose and galactose in liquid culture. The concentrations used were 0.1%, 0.5%, 1%, and 2%. The beta-galactosidase activity was measured in different concentrations of sugar by a protocol described by Miller method (see [20]). The maximum enzyme activities at different concentrations are plotted; the plots are not to scale.

**Remark 5 **The Nyquist plot of a Zames-Falb multiplier is constrained to lie inside a disc in the open right-half *s*-plane, as shown in Fig. 3(ii). In [15], aberrations of monotone nonlinearities, as shown in Fig. 3(iii), are considered and a class of positivity preserving multipliers for these nonlinearities is established. The results of [15] facilitate a class of stabilizing controllers for systems featuring such nonlinearities. It turns out that *Kluyveromyces lactis *exhibits such a nonlinearity when the input is galactose and the output of interest is the GAL4 expression (see Fig. 4).

## Results and discussion

### Determination of the domain of attraction for equilibrium gene expression states

We now establish sufficiency conditions under which a polytope  ≐ {*α*_*i*_*x *≤ 1 | *i *= 1, 2... *n *+ 1} belongs to the domain of attraction of the equilibrium point *x *= 0 given that the state feedback *u *= *Kx *is used to control the galactose entering the cell. Let *ν*_*i *_denote the vertices of . The following result is well known (see [16]).

**Theorem 1 ***Given a closed set E *⊂ ℝ^*n *^*such that the equilibrium point x*_*o*_*is contained in E, suppose the following conditions are satisfied: (i) E is an invariant set of the given system; and, (ii) a Lyapunov function V *(*x*) *exists such that V *(*x*) *is positive definite on E and, further*, *is negative definite along the trajectories of the given system in E. Then, E is an estimate of the domain of attraction of x*_*o*_.

The above theorem can be specialized to our system as follows.

**Theorem 2 ***is in the domain of attraction of an equilibrium point x *= 0 *of **if there exist scalars γ *∈ (0, 1), *c *> 0, *a symmetric positive definite matrix P *∈ ℝ^*n*×*n*^, *and a matrix K such that*(4)(5)

*where Herm*(·) *denotes the Hermitian of *(·). *The desired controller is given by u *= *Kx*.

**Proof: **Our proof uses the results derived in [17] and [18], and can be sketched as follows. Let us consider the function *V *(*x*) = *x*^*T*^*P*^-1^*x *as the candidate Lyapunov function. Since *P *is a symmetric positive definite matrix, *ν*(*x*) is positive definite. It needs to be shown that  is negative definite along the system trajectories on . Observe that the inequality (5) holds not only for the vertices *ν*_*i *_but for all points *x *inside the scaled polytope  since the function on the left-hand side is an affine function of *x*. It can be observed that the left hand side of this inequality is  along the trajectories of  so that  is indeed a Lyapunov function for . We next show that the polytope  contains a level curve of the chosen Lyapunov function. It is well known that the ellipsoid ℰ ≐ {*x *∈ ℝ^5 ^| *x*^*T*^*P*^-1^*x *≤ *c*}contains the polytope  (see [[19], pp. 69]). Now, the polytope  can be expressed as  = {*x *∈ ℝ^5^|*γa*_*i*_*x *≤ 1 *i *= 1, 2, ..., 5}. Now, using the Schur complement, the condition (4) can be re-written as  Hence, by [[19], pp. 70], it follows that  contains ℰ. Hence *V *(*x*) is a Lyapunov function on ℰ. Further, the boundary of ℰ is a level curve of *V *(*x*) whence ℰ is an invariant set. Hence, by Theorem 1, ℰ ⊃  is an estimate of the domain of attraction. Hence the proof. QED.

**Remark 6 **Theorem 2 establishes a lower bound  on the domain of attraction of an equlibrium point and also yields a full-state feedback controller *u *= *Kx *which asymptotically drives a state within  to the equilibrium point. The result applies only for the special case wherein the equilibrium point *x*_*o*_is the origin, and can be extended to cover the case of other equilibrium points.

**Remark 7 **The domains of attraction of the equilibrium points have been experimentally reported as the regions of persistent and non-persistent memory in [1]. Theorem 2 characterizes the domain of attraction for the special case in which a linear time-invariant feedback from the expressed genes is used to control the input galactose.

### Stabilizing feedback controller for gal4 expression in kluyveromyces lactis

If the objective is to control only GAL4 expression, as opposed to controlling *all *individual gene expression levels, the classical multiplier theory might provide a wide range of linear and nonlinear stabilizing controllers. We have experimentally observed that the GAL4 expression exhibits an aberration of monotone nonlinearity when the cell is excited with galactose (see Fig. 4); the expression is further inhibited in the presence of glucose. Some experimental set-ups require that the galactose be injected in a cell such that the GAL4 expression is regulated to a desired value. For these applications, a class of stabilizing controllers may be obtained as follows using the framework of [15]. Let *N *denote this nonlinearity, and let Δ denote the dip in the nonlinearity (see Fig. 4). Let *C *be the controller to be designed. Then, feedback system Σ_*R *_of interest is as follows: *y*_1 _= *N *(*u*_1_), *u*_1 _= *C*(*e*_1_), *e*_1 _= *r *- *y*_1_. Using Theorem 1 of [15], the following result is readily established.

**Lemma 3 ***Let *ℳ_*R *_*denote the class of convolution operators, either continuous-time or discrete-time, such that the impulse response of an M *∈ ℳ_*R *_*is of the form*

*where g, h*(·) ∈ ℝ. *Then *Σ_*R *_*is finite-gain stable if C *∈ ℳ_*R*_.

**Proof: **The proof follows as a ready consequence of [[15], Theorem 1].

**Remark 8 **This controller can be used to control the expression of GAL4. However, it cannot control the cellular memory since it cannot regulate the expression of other genes.

## Conclusion

We have derived an ODE model of the GAL regulatory network of *Saccharomyces cerevisiae*. We have shown that although the ODE model of [4] gives an elegant explanation of the transient response of a subset of this network, it does not exhibit bistability, a key property of the GAL regulatory network. By including more chemical reactions in the approach of [4], we have proposed a 5-state quadratic model of the GAL regulatory network. For this model, we have established sufficiency conditions for the domain of attraction of an equilibrium point for the special case of full-state feedback control. This result is useful in characterizing the persistence of cellular memory. We have experimentally observed that the GAL4 expression in *Kluyveromyces lactis *exhibits an aberration of monotone nonlinearity. For a simplified model of this system, wherein the input is galactose and the output is GAL4 expression, we have derived a class of stabilizing controllers using the results of [15]. Unlike the existing literature on GAL regulatory systems, our approach is not limited to 2 state-variables or 2 parameters; our LMI conditions scale well to address more state-variables and parameters, as is the case in the GAL regulatory system, and can be easily implemented in software.

## Competing interests

The authors declare that they have no competing interests.

## Authors' contributions

The experimental results for *Kluyveromyces lactis *were obtained by Prof. Venkatesh Kareenhalli and Pushkar Malakar. Lemma 1 was derived by Prof. Ganesh Viswanathan and Prof. Vishwesh Kulkarni. Lemma 2 was derived by Prof. Michael Safonov. Theorem 2 was derived by Prof. Vishwesh Kulkarni. The ODE model was derived by Pushkar Malakar and Prof. Vishwesh Kulkarni. The application of [15] is due to Prof. Vishwesh Kulkarni, Prof. Lucy Pao, and Prof. Michael Safonov.

**Proof of Lemma 1: **At an equilibrium, the following equalities hold:

The steady state solution is given by

So, the Jacobian at the steady state (*R*_*ss*_, *Gal*_*ss*_) is given by

As the Jacobian is non-singular for all values of the model parameters, it satisfies the implicit function theorem. As a result, the system cannot exhibit any steady state bifurcations and, therefore, does not exhibit bistability. Next, we investigate the presence of a Hopf bifurcation point that may lead to oscillatory solution or dynamic equilibrium. Now, the model exhibits Hopf bifurcation if and only if trace(*J*) = 0 and det(*J*) *>*0. Hence, the given system has a Hopf bifurcation if and only if

Since *K*_1 _is positive, a Hopf bifurcation exists if and only if

After simplifying the above equations, we get(6)

Hence, Hopf bifurcation exists if and only if (6) holds together with the following equation:

Since all parameters are strictly positive and since *R*_0_, the initial quantity of GAL4p, and *S*_0_, the initial quantity of galactose, are non-zero quantities (see [4]), the above condition cannot be satisfied. Therefore, Hopf bifurcation does not exist for . QED.   □

## Supplementary Material

Additional file 1 The single-page PDF file contains the proof of Lemma 1. Name of the file: APBC2010-ID151-Appendix.pdf. Click here for file
