# Supplementary material for: Stability analysis of the GAL regulatory network in Saccharomyces cerevisiae and Kluyveromyces lactis
Source: BMC Bioinformatics. 2010 Jan 18;11(Suppl 1):S43. doi: 10.1186/1471-2105-11-S1-S43 (PMC3009516; doi:10.1186/1471-2105-11-S1-S43)
Supplement: Additional file 1 — The single-page PDF file contains the proof of Lemma 1. Name of the file: APBC2010-ID151-Appendix.pdf. [file 1471-2105-11-S1-S43-S1.pdf]

Proof: At an equilibrium, the following equalities hold:

$$\begin{aligned}(K_3-K_2)GalR - K_3R_0Gal - (K_3S_0+K_1)R + R_0(K_3S_0+1) &= 0\\ (K_3-K_2)GalR + K_3S_0R_0 - K_3S_0R - K_3R_0Gal &= 0.\end{aligned}$$

The steady state solution is given by

$$R_{ss} = \frac{R_0}{K_1}, \quad Gal_{ss} = \frac{(K_3S_0+K_1)\frac{R_0}{K_1} - R_0(K_3S_0+1)}{(K_3-K_2)\frac{R_0}{K_1} - K_3S_0}.$$

So, the Jacobian at the steady state (R_{ss}, Gal_{ss}) is given by

$$J = \begin{bmatrix}(K_3-K_2)R_{ss} - K_3R_0 & (K_3-K_2)Gal_{ss} - (K_3S_0+K_1)\\ (K_3-K_3)R_{ss} - K_3R_0 & (K_3-K_2)Gal_{ss} - K_3S_0\end{bmatrix}$$

As the Jacobian is non-singular for all values of the model parameters, it satisfies the implicit function theorem. As a result, the system cannot exhibit any steady state bifurcations and, therefore, does not exhibit bistability. Next, we investigate the presence of a Hopf bifurcation point that may lead to oscillatory solution or dynamic equilibrium. Now, the model exhibits Hopf bifurcation if and only if trace$(J) = 0$ and det$(J) > 0$. Hence, the given system has a Hopf bifurcation if and only if

$$(K_3-K_2)R_{ss} - K_3R_0 + (K_3-K_2)Gal_{ss} - K_3S_0 = 0, \quad \text{and} \quad ((K_3-K_1)R_{ss} - K_3R_0)K_1 > 0.$$

Since K_1 is positive, a Hopf bifurcation exists if and only if

$$(K_3-K_2)R_{ss} - K_3R_0 = -\left[(K_3-K_2)Gal_{ss} - K_3S_0\right] > 0 \quad \Rightarrow \quad (K_3-K_2)Gal_{ss} < K_3S_0.$$

After simplifying the above equations, we get

$$\frac{K_3}{K_2K_1}\left(1 - \frac{S_0}{R_1}\right) > 1. \tag{6}$$

Hence, Hopf bifurcation exists if and only if (6) holds together with the following equation:

$$\left[(K_3-K_2)\frac{R_0}{K_1} - K_3R_0\right]^2 + K_2K_3S_0R_0 = 0.$$

Since all parameters are strictly positive and since R_0, the initial quantity of GAL4p, and S_0, the initial quantity of galactose, are non-zero quantities (see [4]), the above condition cannot be satisfied. Therefore, Hopf bifurcation does not exist for $\mathcal{S}_L$. QED. □
